# Knowledge and attitude regarding hepatitis B virus infection and vaccine among hospital patients

**DOI:** 10.1186/2047-2994-4-S1-P257

**Published:** 2015-06-16

**Authors:** SS Katpattil

**Affiliations:** 1 Public Health Dentistry, Yenepoya University, Mangalore, India

## Introduction

Hepatitis B is a potentially life-threatening infection caused by the hepatitis B virus. It is the most serious type of viral hepatitis. About 400 million people have the virus, with most of these people living in Asia. Clearly, this is a significant public health and medical problem.

## Objectives

With this background, the study was conducted to evaluate knowledge and attitude regarding HBV (Hepatitis B virus) infection and its vaccine among the patients attending tertiary care hospital.

## Methods

A Cross-sectional study was done among 856 patients attending a tertiary care hospital, at Mangalore, India, from November 2010 to May 2011after approval from the institutional ethical committee. A pretested structured questionnaire was used to measure the participants’ knowledge and attitude regarding HBV (Hepatitis B virus) infection and its vaccine after obtaining informed consent.

## Results

In all, 856 patients (698 male and 158 female) were studied. 50% of those who were aware had no knowledge about route of transmission, infectivity, or importance of vaccination. Educated individuals were more aware about hepatitis B vaccine (P< 0.05). The percentage of vaccination was 25% among study subjects. Lack of awareness was the common reason for non - vaccination (50%); of them.

## Conclusion

Knowledge of Hepatitis B disease and vaccine was low and misconceptions were common. About One third of the population are vaccinated for hepatitis B. Emphasis should especially be laid on awareness campaigns to educate the public that hepatitis B is vaccine preventable disease. Knowledge of the hepatitis B disease may be useful in determining health care interventions strengthening community-based care for patients.

## Disclosure of interest

None declared.

